# Knockdown of Methylation‐Related Gene MBD2 Blocks Cell Growth by Upregulating p21 Expression in Head and Neck Squamous Cell Carcinoma

**DOI:** 10.1002/cnr2.70080

**Published:** 2024-12-16

**Authors:** Ting Cao, Xia Shen, Fei Pei, Taogeng Jiang, Jun Zhang, Hong Zhou

**Affiliations:** ^1^ Center for Medical Research and Innovation, Shanghai Pudong Hospital Fudan University Pudong Medical Center Shanghai China; ^2^ Department of Otolaryngology, Shanghai Pudong Hospital Fudan University Pudong Medical Center Shanghai China; ^3^ Department of Otolaryngology, Shidong Hospital, Yangpu District Shidong Hospital Affiliated to University of Shanghai for Science and Technology Shanghai China

**Keywords:** 5‐azacytidine (5‐Aza), head and neck squamous cell carcinoma (HNSCC), methyl‐CpG‐binding domain 2 (MBD2), p21, prognosis

## Abstract

**Background:**

Methyl‐CpG‐binding domain 2 (MBD2) attaches to methylated DNA, which mediates methylated gene transcription, leading to gene silencing and affecting tumor progression. The molecular mechanisms of MBD2 in head and neck squamous cell carcinoma (HNSCC) remain insufficiently characterized.

**Aims:**

This study sought to assess the clinical relevance of MBD2 expression in HNSCC, with a particular focus on elucidating its functional role in tumor progression and its regulatory influence on p21 expression and cellular proliferation.

**Methods:**

We analyzed the relationships between MBD2 expression, clinicopathological features, and survival outcomes in HNSCC patients using data from the UALCAN, TCGA, and cBioPortal databases. The functional role of MBD2 in HNSCC was further investigated through in vitro experiments. p21 expression was assessed using western blotting and qRT‐PCR in TU212 and AMC‐HN8 cells. These cells were treated with either shRNA targeting MBD2, 5‐azacytidine (5‐Aza), or a combination of shRNA MBD2 and 5‐Aza. Additionally, cell proliferation and viability were measured in each treatment group.

**Results:**

MBD2 was found to be frequently overexpressed in HNSCC tissues, and its altered expression was significantly associated with reduced overall survival (OS) and disease‐free survival (DFS). Both shRNA‐mediated MBD2 knockdown and 5‐Aza treatment increased p21 expression in HNSCC cells, exhibiting similar functions with additive effects. Furthermore, both treatments significantly inhibited cell proliferation and viability.

**Conclusion:**

These results indicated that shRNA‐mediated MBD2 knockdown suppresses HNSCC cell growth by upregulating p21 expression. In addition to its role as an oncogene, MBD2 may serve as a prognostic biomarker and therapeutic target for HNSCC patients.

## 1 Introduction

Head and neck squamous cell carcinoma (HNSCC) ranks as the seventh most prevalent cancer globally, affecting regions such as the oral cavity, larynx, pharynx, hypopharynx, nasal cavity, and salivary glands [1]. Annually, HNSCC contributes to 890 000 new instances and 450 000 fatalities worldwide, comprising about 4.5% of all cancer diagnoses and fatalities [2]. The incidence of HNSCC is on the rise due to various risk factors like tobacco use, alcohol consumption, betel nut chewing, and infection with the Epstein–Barr virus (EBV) and the human papillomavirus (HPV) [3]. Despite recent progress in surgical interventions, radiotherapy, chemotherapy, and emerging targeted therapies, the 5‐year survival rate of HNSCC patients is still quite low [4]. The underlying molecular mechanisms and prognostic factors of HNSCC remain inadequately understood [5]. Therefore, exploring the possible mechanisms of HNSCC and identifying specific biomarkers or promising therapeutic targets are still urgent and necessary.

DNA methylation is an epigenetic modification and tumor suppressor gene hypermethylation is an important initial event in many malignant tumors [6]. In some cases, it is the only mechanism for tumor suppressor gene inactivation, which is intimately related to the occurrence and progression of cancer [7]. Methyl‐CpG‐binding domain 2 (MBD2) performs a crucial role in tumor progression. It can bind with methylated DNA to form a stable transcription inhibition complex, which inhibits methylated gene transcription, leading to gene silencing and affecting tumor progression. MBD2 has been linked to various cancers in human, including ovarian carcinoma, renal cell carcinoma, hepatocellular carcinoma, colorectal carcinoma, lung adenocarcinoma, and cervical carcinoma. MBD2 is a potential drug target and poor prognosis biomarker in multiple carcinoma [8, 9]. However, in lung adenocarcinoma, MBD2 inhibits the malignant characteristic [10]. A study shows that MBD2 expression is significantly reduced in cervical cancer [11]. All of the above evidence highlights a crucial role of MBD2 in cancer progression. Nevertheless, the precise role and regulatory mechanism of MBD2 in HNSCC remain largely unclear.

DNA methylation modifications, common in human tumors, can be reversed by DNA methyltransferase inhibitors (DNMTIs) [12]. 5‐Azacytidine (5‐Aza), a demethylating agent, integrates into nucleic acids as an analogue of cytidine that DNA methyltransferases (DNMTs) cannot methylate. To date, the US Food and Drug Administration (FDA) has granted 5‐Aza for treating myelodysplastic syndrome (MDS) [13]. Recently, an in vitro study clearly clarified that 5‐Aza and trichostatin A (TSA) can potentiate cell apoptotic responses and DNA damage reactions in allograft squamous cell carcinoma while decreasing tumor invasion, suggesting potential applications for 5‐Aza in HNSCC treatment [14].

Given the evidence of its involvement in other malignancies, understanding how MBD2 contributes to HNSCC progression could provide valuable insights into potential therapeutic approaches. This study examined the prognostic significance of MBD2 in HNSCC, revealing that high MBD2 expression correlates with poor prognosis in patients. According to our previous findings, demethylation of the p21 gene can lead to cell apoptosis in laryngeal squamous cell carcinoma (LSCC) [15]. Furthermore, another aim of the current research was to evaluate the potential function of MBD2 and 5‐Aza in regulating p21 in HNSCC cell lines, which provided evidence that blockade of MBD2 and application of 5‐Aza inhibit cell proliferation in vitro. By examining the interplay between MBD2 and 5‐Aza, this research provides new insights into therapeutic strategies for HNSCC, offering potential avenues for targeted interventions.

## 2 Materials and Methods

### 2.1 Bioinformatic Analysis

The mRNA expressions of MBD2 across various cancer types were analyzed using UALCAN (http://ualcan.path.uab.edu/), an interactive web portal for detailed analysis of the Cancer Genome Atlas (TCGA) gene expression data. The differential expression module in UALCAN generated boxplots to display MBD2 expression across different cancers [16]. UALCAN also facilitated the analysis of MBD2 expression compared to normal tissues and its association with clinicopathological features of patients including age, gender, race, grade, stage, nodal metastasis status, HPV infection status, HPV/p16 infection status, and TP53 mutation status.

The MBD2 expression level of HNSCC was obtained from the Genomic Data Commons (https://portal.gdc.cancer.gov/) [17]. TCGA was chosen as the program (workflow type: HTSeq‐FPKM). Data generated by high‐throughput sequencing and prestandardized were used.

Survival data and mRNA expression alterations of MBD2 in HNSCC were accessible via cBioportal (http://www.cbioportal.org) [18, 19]. The cBioPortal provides an open‐access resource for cancer genomics data, allowing researchers to explore the relationship between genetic alterations and clinical outcomes.

### 2.2 Cell Cultures and Treatments

Human HNSCC cell lines TU212 and AMC‐HN8 were procured from Shanghai Fuheng Biology Corporation and authenticated by STR profiling. No mycoplasma contamination was observed under a microscope. These cells were grown in RPMI 1640 medium (Gibco BRL, Catalog No.11875093) supplemented with 10% fetal bovine serum (FBS, Catalog No.10099‐141), 100 U/mL penicillin, and 100 μg/mL streptomycin in a 5% CO_2_ atmosphere at 37°C. Cells were maintained in the logarithmic growth phase, ensuring 95%–100% viability. For TU212, the initial cell density on seeding for CCK‐8 assay is 3000; and for AMC‐HN8, the number of seeding is 8000. Cells were cultured at low density for 24 h before treatment and transferred to a culture medium containing 0–5 μM 5‐Aza (Sigma‐Aldrich, USA, Catalog No. A2385). An equal volume of PBS containing 0.1% dimethyl sulfoxide is used as vehicle controls.

### 2.3 Transfection

To suppress MBD2 expression, the short hairpin RNA (shRNA) plasmid targeting human MBD2 and the negative control plasmid were acquired from GeneChem company (Shanghai, China, plasmid vector number: GV248). Lipofectamine 3000 (Invitrogen, USA, Catalog No. L3000008) was used for transient transfections in accordance with the guidelines provided by the manufacturer. After transfection, cells were cultured for 24–48 h before subsequent trials.

### 2.4 Quantitative Reverse Transcription Polymerase Chain Reaction (qRT‐PCR)

Total RNA was sequestered using TRIzol reagent (Invitrogen, Catalog No.15596026CN). One microgram of total RNA was reverse transcribed using the QuantiNova Reverse Transcription Kit (QIAGEN, Catalog No. 205411) in compliance with the manufacturer's guidelines. qRT‐PCR was conducted to measure the expression of specific genes using the QuantiNova SYBR Green PCR Kit (QIAGEN, Catalog No. 208052). The relative gene expression was determined by using the 2^−ΔΔCt^ method with GAPDH as the reference gene. Primer sequences are listed in Table 1.

**TABLE 1:** Primers used in the current study.

Gene	Primers (5′–3′)
MBD2‐F	CCATGGAACTACCCAAAGGTCTT
MBD2‐R	CAGCAGATAAAAGGGTCTCATCATT
MBD4‐F	TCTAGTGAGCGCCTAGTCCCAG
MBD4‐R	TTCCAATTCCATAGCAACATCTTCT
DNMT1‐F	CCTGTACCGAGTTGGTGATGGT
DNMT1‐R	CCTTCCGTGGGCGTTTC
p21‐F	CTCCTTCCCATCGCTGTCAC
p21‐R	TCACCCTGCCCAACCTTAGA
DNMT3a‐F	CTCCTGTGGGAGCCTCAATGTTACC
DNMT3a‐R	CAGTTCTTGCAGTTTTGGCACATTCC
DNMT3b‐F	ACCACCTGCTGAATTACTCACGC
DNMT3b‐R	GATGGCATCAATCATCACTGGATT
GAPDH‐F	TGCCCTCAACGACCACTTT
GAPDH‐R	GGTCCAGGGGTCTTACTCCTT

Abbreviations: DNMTs, DNA methyltransferases; F, forward; MBDs, methyl‐CpG‐binding domain; R, reverse.

### 2.5 Western Blot Analysis

Cell lysis was performed in cold RIPA buffer (Thermo Scientific, USA, Catalog No. 89901) with the addition of phenylmethylsulfonyl fluoride (PMSF), followed by protein concentrations measurement using the BCA assay (Thermo Scientific, Catalog No. 23227). Equal amounts of obtained protein were subsequently separated using SDS‐PAGE and transferred to a 0.45‐μm PVDF membrane (Bio‐Rad, USA, Catalog No. 1620264). The membrane was used to block with 5% nonfat milk (Amresco, USA, Catalog No. 97063‐958) and incubated with primary antibodies against MBD2 (1:1000, Proteintech, Catalog No. 55200‐1‐AP), p21 (1:1000, CST, Catalog No. 2947S), and β‐actin (1:1000, CST, Catalog No. 4970S). Following primary antibody incubation, the membrane was treated with horseradish peroxidase (HRP)‐conjugated secondary antibodies (1:4000, CST, Catalog No. 7074S). Protein bands were visualized using OmegaLumG (Aplegen, USA).

### 2.6 Cell Proliferation and Viability Assay

Cells were placed in 96‐well plates and grown for the specified durations. After the addition of CCK8 solution (Dojindo Molecular Technologies, Japan, Catalog No.CK04), the cells were incubated for 2 h before measuring the absorbance at 450 nm (OD 450) using a microplate reader (Infinite 200pro, TECAN, Switzerland). Cell viability was determined by trypan blue staining with a Vi‐Cell (Beckman Coulter, USA). Each experiment was conducted at least three times in triplicate.

### 2.7 Statistical Analysis

Statistical analyses were carried out using SPSS 22.0 software (SPSS Inc., Chicago, IL, USA) and Prism software (GraphPad, La Jolla, CA, USA). Results from at least three separate trials were expressed as the mean ± SEM. The Chi‐squared (*χ* ^2^) test was used to evaluate the relationship between MBD2 expression and clinicopathological features of HNSCC. The Kaplan–Meier method and the log‐rank test were used to estimate overall survival (OS) and disease‐free survival (DFS). Bonferroni's or Tukey's test following significant two‐way repeated measures ANOVA was applied to compare the effects of 5‐Aza and shMBD2 treatments on cell proliferation. Two‐tailed student's *t*‐test assessed MBD2 expression after shRNA transfection in two cell lines. One‐way ANOVA followed by Tukey's post hoc test compared the expression of methylation‐related genes, cell viability, and p21 mRNA and protein expression. Statistically significant was considered for *p* values < 0.05.

## 3 Results

### 3.1 Relationship Between MBD2 mRNA Expression Alterations and Prognosis in HNSCC Patients

**FIGURE 1:**
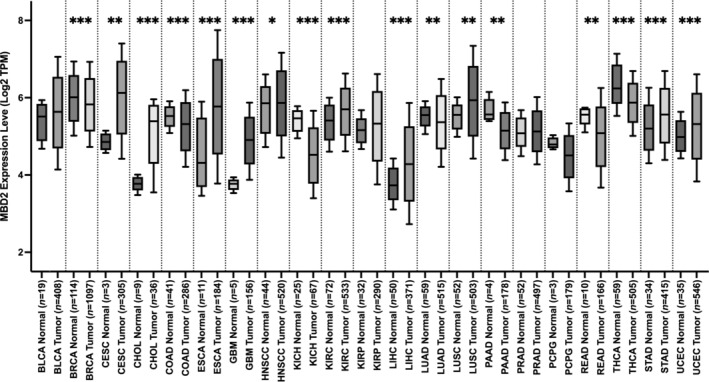
The expression of MBD2 in diverse human cancers. MBD2 expression levels in various types of cancers were analyzed using UALCAN. Data were presented as box‐and‐whisker plots. *p < 0.05, **p < 0.01, ***p < 0.001.

**FIGURE 2:**
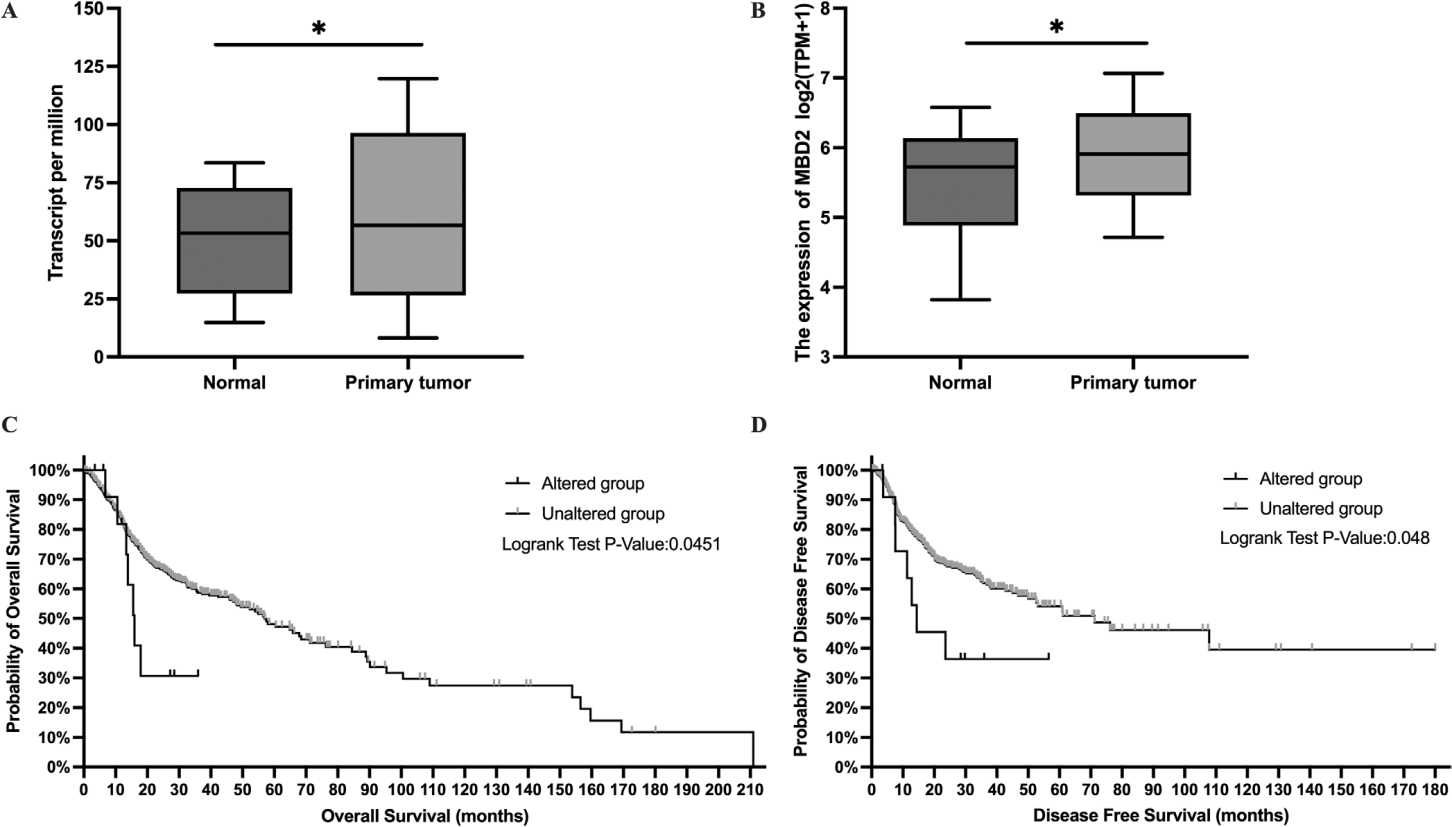
Relationship between MBD2 mRNA expression alterations and prognosis in HNSCC patients. MBD2 expression in HNSCC samples was remarkably higher than in adjacent normal samples from TCGA (A). MBD2 levels in HNSCC tissues were markedly elevated compared to paired paracarcinoma tissues from TCGA (B). Prognostic analysis using the cBioPortal database indicated that altered MBD2 expression correlates with poor OS (C) and DFS (D) in HNSCC patients. Data were presented as box‐and‐whisker plots. *p < 0.05.

Using UALCAN, we found that MBD2 mRNA is highly expressed in several cancers types, including cervical squamous cell carcinoma and endocervical adenocarcinoma (CESC), cholangiocarcinoma (CHOL), esophageal carcinoma (ESCA), glioblastoma multiforme (GBM), kidney renal clear cell carcinoma (KIRC), liver hepatocellular carcinoma (LIHC), lung squamous cell carcinoma (LUSC), stomach adenocarcinoma (STAD), uterine corpus endometrial carcinoma (UCEC), and HNSCC, compared to normal samples (Figure 1). To further assess MBD2 expression in HNSCC, we analyzed mRNA data from TCGA, which showed significantly higher MBD2 levels in 520 HNSCC samples compared to 44 adjacent normal samples (*p* = 0.011) (Figure 2A). Further, the same results were verified in HNSCC tissues and paired paracarcinoma tissues (*p* = 0.024) (Figure 2B). Survival data from cBioPortal indicated that altered MBD2 expression is negatively correlated with poor OS and DFS in HNSCC patients (Figure 2C,D).

### 3.2 MBD2 Expression Is Associated With Clinical Parameters in HNSCC

**FIGURE 3:**
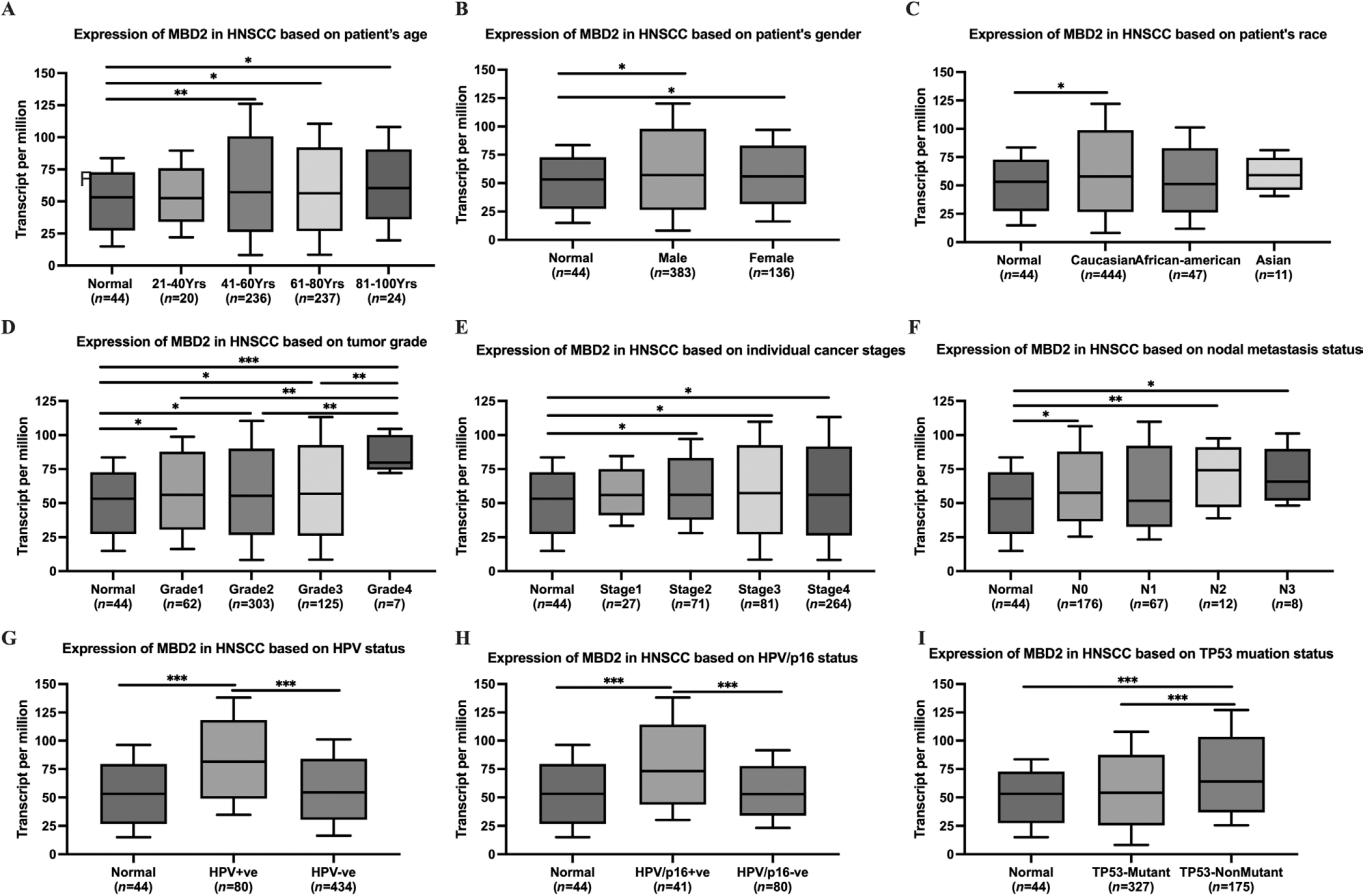
Relationship of MBD2 expression with clinical parameters in HNSCC. MBD2 expression is associated with HNSCC patient age (A), gender (B), race (C), tumor grade (D), cancer stage (E), nodal metastasis status (F), HPV status (G), HPV/p16 status (H), and TP53 mutation status (I). Data were presented as box‐and‐whisker plots. *p < 0.05, **p < 0.01, ***p < 0.001.

We investigated the relationship between MBD2 and clinical parameters in HNSCC patients by utiling the UALCAN database. Patients with HNSCC were categorized according to their age, gender, race, tumor grade, tumor stage, nodal metastasis status, HPV infection status, HPV/p16 infection status, and TP53 mutation status. Significant upregulation of MBD2 expression was observed in the 41–60, 61–80, and 81–100 age groups, with the 61–80 group exhibiting the lowest expression (Figure 3A). No significant difference was found between male and female groups (Figure 3B). Caucasians with HNSCC showed higher MBD2 expression compared to normal individuals (Figure 3C). Significant differences in MBD2 expression were observed between Grade 4 and Grades 1, 2, and 3 (Figure 3D). While MBD2 expression varied significantly between normal and tumor stages or lymph node metastasis statuses, there were no significant differences within these subgroups (Figure 3E,F). Significant differences were also noted in MBD2 levels with HPV infection (Figure 3G) and HPV/p16 infection (Figure 3H). Additionally, MBD2 expression was increased in the TP53 nonmutant group compared to the TP53 mutation group (Figure 3I).

### 3.3 MBD2 Expression and Cell Proliferation in TU212 and AMC‐HN8 Cells

**FIGURE 4:**
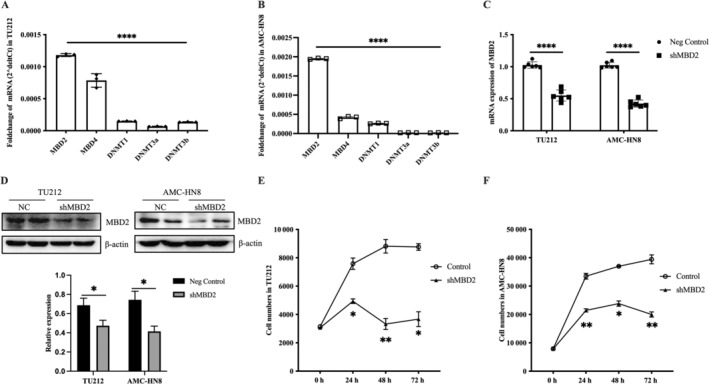
MBD2 expression and cell proliferation in TU212 and AMC‐HN8 cells. (A, B) The mRNA levels of methylation‐related genes (MBD2, MBD4, DNMT1, DNMT3a, and DNMT3b) in TU212 and AMC‐HN8 cells. (C) MBD2 mRNA expression in TU212 and AMC‐HN8 cells 24 h posttransfection, measured via qRT‐PCR. (D) MBD2 protein expression in cells 48 h posttransfection, evaluated by Western blot and normalized using β‐actin. (E, F) At 24, 48, and 72 h, CCK‐8 is used to measure cell proliferation. Data were expressed as mean ± SEM. (A, B) One‐way ANOVA with Tukey's post hoc tests. (C, D) Two‐tailed student's *t*‐test. (E, F) Two‐way repeated measures ANOVA with Bonferroni's post hoc tests. *p < 0.05, **p < 0.01, ****p < 0.0001.

To elucidate the biological function of MBD2 in HNSCC progression, we employed TU212 and AMC‐HN8 cells as experimental models. Initially, we evaluated the expression levels of methylation‐related genes MBD2, MBD4, DNMT1, DNMT3a, and DNMT3b, finding that MBD2 mRNA levels were noticeably higher in these cells compared to the other genes (Figure 4A,B). We then reduced MBD2 expression using shRNA targeting the MBD2 gene, with scrambled shRNA as a control. Transfection efficiency was verified by viewing GFP‐expressing cells using a fluorescence microscope. Both western blot analysis and qRT‐PCR presented an effective reduction of MBD2 protein and mRNA levels in TU212 and AMC‐HN8 cells (Figure 4C,D). Additionally, we found that shRNA MBD2 significantly inhibits cell proliferation in these cell lines (Figure 4E,F).

### 3.4 Effect of 5‐Aza Treatment Alone on p21 Expression, Cell Proliferation, and Viability in TU212 and AMC‐HN8 Cells

**FIGURE 5:**
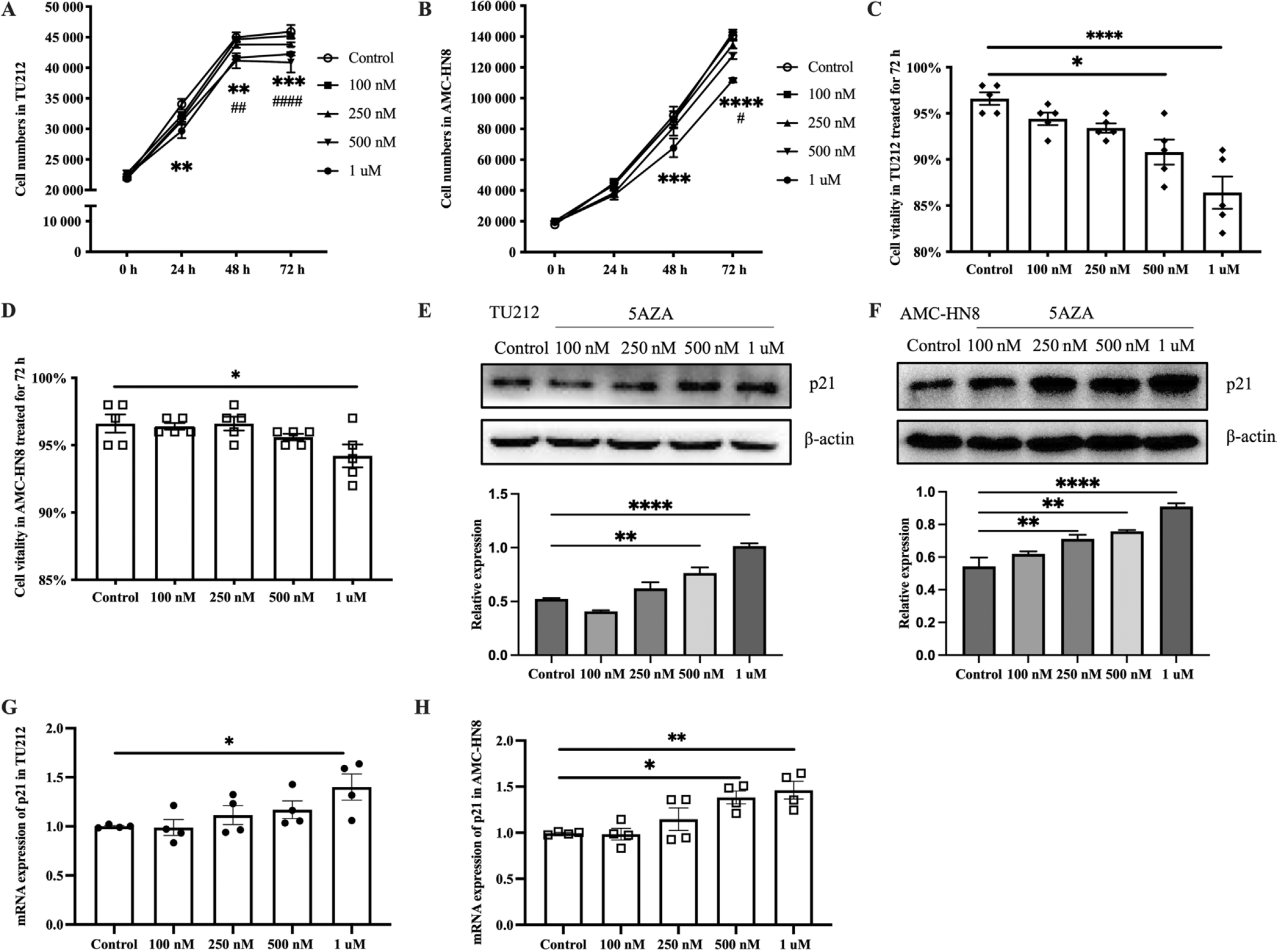
Effect of 5‐Aza treatment alone on p21 expression, cell proliferation, and viability in TU212 and AMC‐HN8 cells. (A, B) At 24, 48, and 72 h, CCK‐8 is used to measure cell proliferation. (C, D) Cell viability was assessed with Vi‐Cell equipment after 72 h of 5‐Aza treatment. (E, F) p21 protein levels were analyzed using Western blot and normalized using β‐actin. (G, H) Relative p21 mRNA levels were measured with qRT‐PCR analysis. Data were expressed as mean ± SEM. (A, B) Two‐way repeated measures ANOVA with Tukey's post hoc tests. *p (control vs. 1 μM group), ^#^ *p* (control vs. 500 nM group). (C–H) One‐way ANOVA with Tukey's post hoc tests. *p < 0.05, **p < 0.01, ***p < 0.001, ****p < 0.0001.

To assess the impact of 5‐Aza on HNSCC cell proliferation and viability, we treated TU212 and AMC‐HN8 cells with varying concentrations of 5‐Aza. The outcomes showed a dose‐dependent inhibitory effect on both cell proliferation and viability (Figure 5A–D). Additionally, we evaluated p21 expression in the treated cells. 5‐Aza significantly and dose‐dependently increased p21 protein levels in both cell lines (Figure 5E,F). qRT‐PCR analysis confirmed increased p21 mRNA expression at higher concentrations of 5‐Aza (Figure 5G,H). The highest p21 expression and strongest inhibitory effects on proliferation and viability were observed at 1 μM 5‐Aza, which was subsequently used for further experiments.

### 3.5 Effect of shRNA MBD2 and 5‐Aza Combination Treatment on p21 Expression, Cell Proliferation, and Viability in TU212 and AMC‐HN8 Cells

**FIGURE 6:**
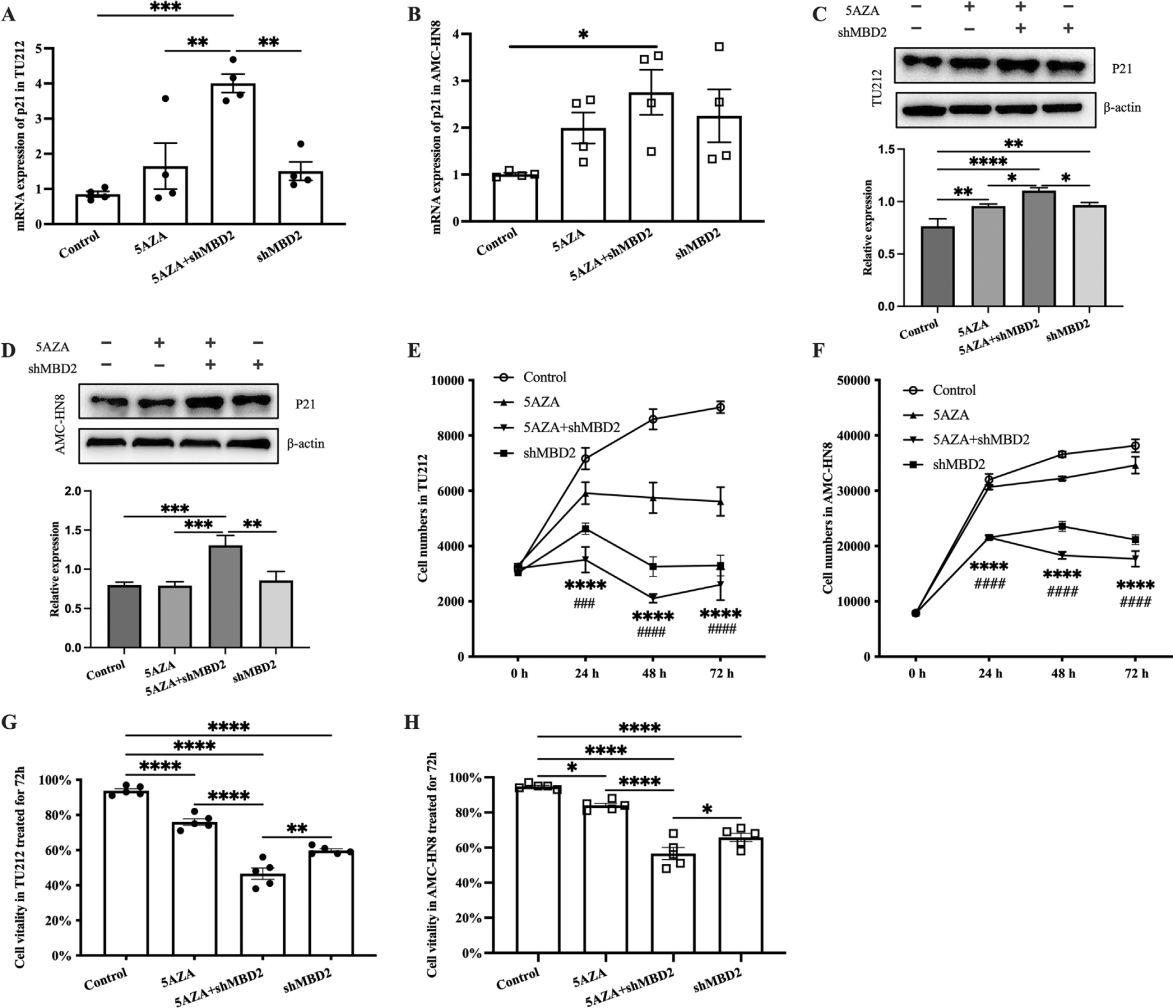
Effect of shRNA MBD2 and 5‐Aza combination treatment on p21 expression, cell proliferation, and viability in TU212 and AMC‐HN8 cells. (A, B) Relative p21 mRNA levels measured with qRT‐PCR after 24‐h transfection followed by 5‐Aza (1 μM) treatment for 72 h. (C, D) Representative p21 protein blots from Western blot analysis of cell lysates and normalized using β‐actin. (E, F) At 24, 48, and 72 h, CCK‐8 is used to measure cell proliferation. (G, H) Cell viability was assessed with Vi‐Cell equipment after 72 h of 5‐Aza treatment. Data were expressed as mean ± SEM. (A–D, G–H) One‐way ANOVA with Tukey's post hoc tests. (E, F) Two‐way repeated measures ANOVA with Tukey's post hoc tests. *p (control vs. shMBD2 and 5‐Aza combination treated group), ^#^ *p* (control vs. shMBD2 treated group). *p < 0.05, **p < 0.01, ***p < 0.001, ****p < 0.0001.

To investigate MBD2's role in inhibiting HNSCC cell growth, we transiently transfected TU212 and AMC‐HN8 cells with shRNA targeting MBD2, followed by 1 μM 5‐Aza treatment for 72 h. Post‐transfection, Western blot analysis, and qRT‐PCR were employed to measure p21 levels. Both shRNA MBD2 and 5‐Aza alone significantly upregulated p21 protein expression, while their combination further increased p21 levels of proteins and mRNA in both cell lines (Figure 6A–D). Cell proliferation and viability assays revealed that shRNA MBD2 or 5‐Aza alone significantly inhibited these parameters, with the combination showing even stronger inhibition (Figure 6E–H). Notably, shRNA MBD2 and 5‐Aza exhibited similar functions with additive effects in combination.

## 4 Discussion

DNA methylation can have a direct or indirect impact on gene transcription and expression without altering in primary sequences and gene products of DNA [20]. Aberrant methylation of tumor‐associated genes occurs in nearly all human tumors, influencing tumorigenesis through chromatin structure and modulation of tumor suppressor genes and oncogenes [21, 22]. DNA methylation regulators include writers like DNMTs, erasers such as 10–11 translocation proteins (TETs), and readers like methyl‐CpG‐binding proteins (MBPs). MBPs like MBD2 recognize methylated CpG islands, typically promoting gene silencing at methylated loci [23]. MBD2, a member of the MBD family, inhibits gene transcription through the recruitment of histone deacetylase complexes such as Sin3A and NuRD/Mi‐2 [24]. It affects various genes involved in tumor regulation such as SFRP [9], Cyclin D1 [10], p53 [10], CDKN1C [25], and Ki‐67 [26], underscoring the pivotal role in abnormal epigenetic tumor regulation. However, the expression pattern and functional roles of MBD2 in HNSCC pathogenesis are still unknown. In our study, we found MBD2 upregulation in cancers including HNSCC, correlating with poor clinicopathological features. Patients with unaltered MBD2 mRNA expression had better OS and DFS outcomes in contrast to those with alterations. Comparing MBD2 with other methylation‐related genes confirms its significant role in HNSCC development and progression, suggesting MBD2 as an independent prognostic factor. The lack of sufficient patient tissue samples to verify the proposed association between MBD2 expression and clinicopathological features in HNSCC represents a limitation of this study. Further future studies need to fully understand the effects of MBD2 on the regulation of HNSCC progression. With further studies of MBD2, future research directions could include exploring its mechanism of action in HNSCC as well as developing prognostic models and therapies based on MBD2 expression levels. These studies will contribute to a better understanding of the role of MBD2 in tumorigenesis and development and provide new strategies for the diagnosis and treatment of HNSCC.

P21, a universal cell cycle regulator downstream of p53, inhibits CDK and cyclin complexes, arresting the cell cycle and suppressing cell proliferation [27, 28]. Various reports have proven that p21 can contribute to tumor suppression by triggering cellular apoptosis [29]. Mutations in p21 are rare but dysregulation via promoter hypermethylation or hypomethylation occurs in several cancers, influencing transcriptional control [30]. In particular, promoter methylation of p21 has been shown to be critical in hematological malignancies [31]. Recently, Zohny et al. found the p21 promoter is hypermethylation in over 50% of breast cancer samples and proposed hypermethylation of the p21 promoter as a diagnostic marker of breast cancer [32]. Our previous study showed that there was hypermethylation of the CpG island of the p21 gene promoter in LSCC tissues and cells. Demethylation of 5‐Aza can induce modification of CpG island demethylation of p21 gene promoter, alter the regulation of tumor cell cycle genes, and promote cell apoptosis [15]. The outcomes of this experimental investigation are in line with the results of previous studies.

To further illuminate the molecular mechanisms of MBD2 in HNSCC, our study assessed the efficacy of shRNA MBD2 and 5‐Aza treatment in TU212 and AMC‐HN8 two HNSCC cell models. The findings of our investigation demonstrated that the downregulation of MBD2 and 5‐Aza inhibits the proliferation and viability of HNSCC cells by upregulating p21 expression. Our study provided preliminary evidence of the synergistic regulation of shRNA MBD2 and 5‐Aza on p21 gene expression and biological function in HNSCC cells. Studies show that p21 silencing is epigenetically regulated not only by DNA methylation but also by other epigenetic regulations and other additional post‐transcriptional mechanisms [30]. Therefore, the specific mechanism of MBD2 on p21 needs to be confirmed experimentally. The precise molecular mechanisms by which MBD2 influences HNSCC cell proliferation, migration, and invasion require further investigation. Further studies will explore the transcription factors involved in the regulation of p21 gene expression, elucidate the specific process of DNA methylation regulation of p21 expression in HNSCC, and establish a new theoretical foundation for the treatment of HNSCC. Future animal studies in vivo are required to establish the plausibility of knockdown MBD2 or overexpression p21 in naked mice that is proposed here to retard cancer progression.

In conclusion, our study strengthens the link between MBD2 and HNSCC, highlights its high expression in tumor tissues and association with poor prognosis and may be an independent prognostic factor for HNSCC patients. shRNA MBD2 inhibits HNSCC cell growth via enhancing the expression of p21. MBD2 promotes cell viability and proliferation in HNSCC cells, acting as an oncogene. Therefore, targeting MBD2 could offer a promising therapeutic strategy for HNSCC treatment, pending further investigation into its precise molecular mechanisms.

## Author Contributions

**Ting Cao:** conceptualization, data curation, funding acquisition, writing – original draft, methodology. **Xia Shen:** data curation, formal analysis, writing – original draft. **Fei Pei:** formal analysis, methodology. **Taogeng Jiang:** formal analysis, validation, investigation. **Jun Zhang:** conceptualization, data curation, writing – review and editing. **Hong Zhou:** conceptualization, writing – review and editing, supervision, project administration.

## Conflicts of Interest

The authors declare no conflicts of interest.

## Data Availability

The data that support the findings of this study are available from the corresponding author upon reasonable request.
